# Analysis of Heat Stress and the Indoor Climate Control Requirements for Movable Refuge Chambers

**DOI:** 10.3390/ijerph13050518

**Published:** 2016-05-20

**Authors:** Xiaoli Hao, Chenxin Guo, Yaolin Lin, Haiqiao Wang, Heqing Liu

**Affiliations:** 1 School of Energy and Safety Engineering, Hunan University of Science and Technology, Xiangtan 411201, China; guochenxing1216@163.com (C.G.); hqwang@hnust.edu.cn (H.W.); hqliu8222638@163.com (H.L.); 2 Hunan Provincial Key Laboratory of Safe Mining Techniques of Coal Mines, Xiangtan 411201, China; 3 School of Civil Engineering and Architecture, Wuhan University of Technology, Wuhan 430070, China; yaolinlin@gmail.com

**Keywords:** movable refuge chamber, heat stress, PHS model, thermal environment, ergonomics

## Abstract

Movable refuge chambers are a new kind of rescue device for underground mining, which is believed to have a potential positive impact on reducing the rate of fatalities. It is likely to be hot and humid inside a movable refuge chamber due to the metabolism of trapped miners, heat generated by equipment and heat transferred from outside. To investigate the heat stress experienced by miners trapped in a movable refuge chamber, the predicted heat strain (PHS) model was used to simulate the heat transfer process between the person and the thermal environment. The variations of heat stress with the temperature and humidity inside the refuge chamber were analyzed. The effects of air temperature outside the refuge chamber and the overall heat transfer coefficient of the refuge chamber shell on the heat stress inside the refuge chamber was also investigated. The relationship between the limit of exposure duration and the air temperature and humidity was numerically analyzed to determine the upper limits of temperature and humidity inside a refuge chamber. Air temperature of 32 °C and relative humidity of 70% are recommended as the design standard for internal thermal environment control of movable refuge chambers.

## 1. Introduction

Coal production is considered a complex and hazardous activity due to its dangerous and dynamic characteristics. Fatalities frequently occur in underground coal mines every year. According to the recent reports from the Mine Safety and Health Administration, the death toll in U.S. coal mine accidents was 16 in 2014 [1]. The Chinese coal industry has been considered as the most hazardous one in the world, though a declining trend can be observed in the absolute number of deaths and the death rate per million tons in recent years in China’s coal mining industry due to a number of significant measures taken by the Chinese government [2]. The death toll in China's coal mines in 2010 was 2433 and the death rate per million tons was 0.76. These numbers were much higher than those of US, which were 48 and 0.049 in the same year, respectively [3,4]. Frequent fatal accidents have a severe negative impact on coal mine production and cause huge economic losses as well [5].

It is urgent to develop new technology to reduce the rate of fatalities in coal mining accidents. Movable refuge chambers, a new rescue device, are believed to have a potential positive impact on reducing the rate of fatalities in underground mining. The investigation results from Ounanian [6] on thirty-eight disasters occurred from 1970 through 2006 indicated that refuge chambers would have had a positive effect on 32% of the disasters and a reduction in the fatality rate of over 29% could have been potentially achieved if refuge chambers had been available when the disasters occurred. The importance of mining shelters in saving lives and decreasing the rates of fatalities and injuries has been recently demonstrated by the successful rescue of thirty-three Chilean miners who had been trapped in a 700-m underground mine for sixty-nine days [7].

The fundamental function of a movable refuge chamber is to provide a protected, secure, respirable, and closed space for trapped miners during emergencies, where miners can stay to await rescue. A refuge chamber should have the capability to protect the trapped miners from the explosive blasts, high temperature fumes and noxious gases resulting from gas and/or coal dust explosions, fires, coal and gas outbursts, *etc.*, in coal mine accidents. It should also provide a respirable atmosphere, non-perishable food, drinking water and a tolerable internal thermal environment to increase the survival rate of trapped miners.

**Figure 1:**
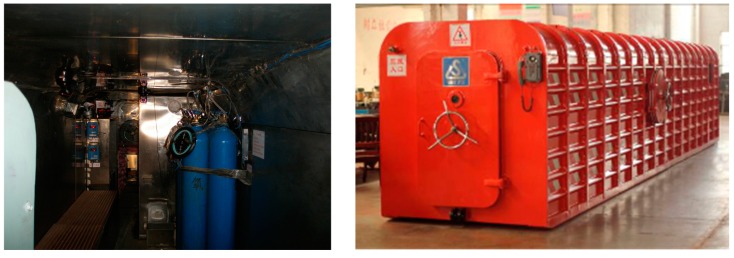
Photograph of a movable refuge chamber, inside (**left**) and outside (**right**).

There are two main types of refuge stations—permanent and temporary—each with varying designs and sizes. Movable refuge chambers, which are ordinarily constructed of steel (see Figure 1), belongs to the temporary refuge station type. Movable refuge chambers have been specially designed to be used in confined working areas and to be moved and relocated quickly and efficiently. Movable refuge chambers have robust construction and are equipped with breathable air supply systems, harmful gas removal systems, power supply systems, air-conditioning systems, lighting systems, monitoring and control systems, communication systems, and waste disposal systems. Non-perishable food, drinking water and a first aid kit are also stored in refuge chambers for life support. Seats are also provided for trapped miners to rest. Movable refuge chambers should have the ability to ensure the survival of their occupants during their protection time without requiring external services.

Due to the novelty of movable refuge chambers, they have attracted much attention in recent years. Fasouletos [8] performed a parametric study on the requirements for designing an optimal refuge chamber. Mitchell [9] examined the capability of refuge chambers to withstand overpressure and the requirements for the internal air and thermal environment of refuge chambers. Meng [10] proposed the basic design principles of a refuge chamber system. The structural safety of refuge chambers is important for the protection of the chamber when an accident, such as gas or dust explosion, coal and gas outburst, and roof collapse, occurs. Any deformation or damage to the chamber shell may decrease its airtightness and it may partially or even completely lose its protective ability. The safe performance and dynamic response of refuge chambers under explosion loads had been experimentally and/or numerically investigated by some researchers [11,12,13,14]. High-quality video and audio contacts between a refuge chamber and surface control center could alleviate the anxiety of the trapped miners and assist in management of the emergency [15]. Zhang [16] and Yao *et al.* [17] proposed methods to improve the quality of images and signals in the communication between refuge chambers and the monitoring and control systems.

Provision of a respirable atmosphere is one of the basic requirements of a refuge chamber. A respirable atmosphere can be achieved by replenishing the oxygen consumed by occupants and scrubbing the excess carbon dioxide and carbon monoxide generated from human metabolic activity and facilities operations. Li *et al.* [18] and Jia *et al.* [19] experimentally investigated the purification characteristics of carbon dioxide and carbon monoxide in the closed space of a refuge chamber with various air scrubbing agents.

Psychological issues are also important factors that should be considered during the design of a refuge chamber. The psychological state of the individual has an effect on his/her cognition, as well as emotional, behavioral, and physical state and thus plays a key role in a successful escape [20]. Margolis *et al.* [21] developed a multimedia training program to teach miners about the potential psychological stress and physiological impacts while being in a refuge chamber, with the purpose to increase the awareness of what it would be like in a practical refuge chamber and to improve the rate of correct response when real emergency occurred. The clinical response and physical discomfort of twenty individuals inside a refuge chamber were measured and analyzed by Mejías *et al.* [7] and it was suggested that the current standards for refuge chambers in Chile need to be revised.

Besides, internal thermal environment control is also a challenge for the design of a movable refuge chamber. The temperature and humidity will rise quickly if no air-conditioning measures are adopted [22]. It is also likely to be hot and humid in a movable refuge chamber and high levels of heat and humidity are harmful, and even dangerous. Excessive heat stress may result in fatalities and injuries even if the toxic gas concentration in the chamber is low. However, there has been little open literature on the quantitative analysis of the heat stress in a movable refuge chamber. In this paper, the predicted heat strain model is used to quantitatively investigate the heat stress experienced by miners trapped in a movable refuge chamber and to determine the upper limits of temperature and humidity for maintaining a tolerable thermal environment in the chamber.

## 2. Predicted Heat Strain Model

To analytically evaluate and interpret the thermal stress experienced by the human body in a hot and humid environment, a mathematical model was developed and validated by Malchaire *et al.* [23], and later adopted by ISO 7933 [24] and called the Predicted Heat Strain (PHS) model. To predict the likely heat strain of body in response to the thermal environment, the PHS model calculates the heat transfer between the person and the thermal environment by using six basic parameters, which are air temperature, humidity, radiant temperature, air velocity, metabolic heat production and clothing insulation. The required evaporative heat flow can be calculated by using Equation (1) according to the thermal equilibrium of the body:

(1) $$E_{\text{req}}=M-W-C_{\text{res}}-E_{\text{res}}-C-R-{\text{dS}}_{\text{eq}}$$

where *E_req_* is the required evaporative heat flow to meet the heat balance of the body, in W/m^2^. *M* is the metabolic rate of the body, in W/m^2^, which can be estimated or measured with the methods described by ISO 8996 [25]. *W* is the effective mechanical power, in W/m^2^, which is small and can be neglected in most situations. *C_res_* is the heat flow by respiratory convection, in W/m^2^. *E_res_* is the heat flow by respiratory evaporation, in W/m^2^-K. *C* and *R* are the convective and radiative heat flows at the skin surface, respectively, in W/m^2^. *dS_eq_* is the body heat storage rate for increase in core temperature associated with the metabolic rate, in W/m^2^. The various terms in Equation (1) can be calculated by Equations (2)–(6), respectively:

(2) $$E_{\text{res}}=0.00127M\left(59.34+0.53t_{a}-11.63P_{a}\right)$$

(3) $$C_{\text{res}}=0.00152M\left(28.56-0.885t_{a}+0.641P_{a}\right)$$

(4) $$C=h_{\text{cdyn}}\times f_{\text{cl}}\times \left(t_{\text{cl}}-t_{a}\right)$$

(5) $$R=h_{r}\times f_{\text{cl}}\times \left(t_{\text{cl}}-t_{r}\right)$$

(6) $${\text{dS}}_{\text{eq}}=c_{\text{sp}}\left(t_{\text{cr},\text{eq},i}-t_{\text{cr},\text{eq},i-1}\right)\left(1-\alpha \right)$$

where *t_a_* is the dry-bulb temperature, in °C. *t_r_* is the mean radiant temperature of the environment, in °C. *t_cl_* is the clothing surface temperature, in °C. *t_cr,eq_* is the equilibrium core temperature of body, in °C, which is a function of metabolic rate. *P_a_* is the partial water vapor pressure in the ambient air, in kPa. *h_cdyn_* is the dynamic convective heat transfer coefficient between the clothing and the ambient air, in W/m^2^-K. *h_r_* is the radiative heat transfer coefficient between the clothing and the surrounding surface, in W/m^2^-K. *f_cl_* the clothing area factor, dimensionless. *c_sp_* is the specific heat of the body, which is about 3470 J/kg-K. α is the fraction of the body mass at the skin temperature.

The required sweat rate and the required skin wettedness can be calculated by Equations (7) and (8), respectively:

(7) $${\text{SW}}_{\text{req}}=E_{\text{req}}/\eta$$

(8) $$w_{\text{req}}=E_{\text{req}}/E_{\text{max}}$$

where η is the evaporative efficiency of sweating, dimensionless. *E_max_* is the maximum evaporative heat flow at skin surface, in W/m^2^. *SW_req_* is the required sweat rate, in W/m^2^. *w_req_* is the required skin wettedness, dimensionless.

Rectal temperature can be derived from the heat storage rate of the body, which can be calculated by the following equations:

(9) $$S=E_{\text{req}}-E_{p}+S_{\text{eq}}$$

(10) $$t_{\text{cr},i}=\frac{1}{1-\frac{\alpha_{i}}{2}}\left(\frac{S_{i}A_{\text{Du}}d\tau }{c_{\text{sp}}W_{b}}+t_{\text{cr},i-1}-\frac{t_{\text{cr},i-1}-t_{\text{sk},i-1}}{2}\alpha_{i-1}-t_{\text{sk},i}\frac{\alpha_{i}}{2}\right)$$

(11) $$t_{\text{re},i}=t_{\text{re},i-1}+\frac{2t_{\text{cr},i}-1.962t_{\text{re},i-1}-1.31}{9}$$

where *E_p_* is the predicted evaporative heat flow, in W/m^2^. *S_eq_* is the body heat storage rate due to the increase of core temperature associated with metabolic rate, in W/m^2^. *A_Du_* is the DuBois body surface area, in m^2^. *t_cr_*, *t_re_* and *t_sk_* are, respectively, the core temperature, the rectal temperature and the mean skin temperature of body, in °C. *W_b_* is the body mass, in kg. *dτ* is the time interval between the time *i* and time *i − 1*, in min. The subscript *i* and *i − 1* mean the values at time *i* and time *i − 1*, respectively. Equations (10) and (11) describe the relationships between the core temperature and the rectal temperature of body and the exposure time in hot and humid environment, respectively.

The PHS model provides a method for predicting the core temperature and sweat rate that the human body will develop in response to the hot working conditions. Two physiological criteria, maximum skin wittedness and maximum sweat rate, are used to predict the core temperature and sweat rate. Two heat strain criteria, maximum acceptable rectal temperature and maximum allowable water loss, are used to determine the maximum allowable exposure time of human body in hot environment. A rectal temperature limit of 38 °C and a maximum water loss of 5% of body mass for 95% of the working population are recommended by ISO 7933 [24]. More details about the PHS model can be found in the paper by Malchaire *et al.* [23] and the ISO 7933 norms [24].

The main purposes of ISO 7933 [24], which is based on the PHS model, include: (a) the evaluation of the thermal stress in conditions likely to lead to excessive core temperature increase or water loss for the standard subject [24]; (b) the determination of the maximum allowable exposure time under given hot working conditions [24]; (c) the determination of the environmental climate parameters to ensure a standard subject can work or stay in the environment for the demanded exposure time without physical damage [26]. In this paper, the PHS model was used to evaluate the heat stress experienced by trapped miners in a hot and humid environment of the refuge chamber and to determine the upper limits of temperature and humidity inside the refuge chamber to protect workers from heat stress, dehydration and collapse.

By using the PHS model, the heat stress experienced by trapped miners in a movable refuge chamber can be analytically evaluated with the internal thermal environment parameters. When a mine disaster occurs, miners are trained to enter the refuge chamber as quickly as possible and wait for rescue if they cannot escape. The metabolic rate of the body at rest in a thermos neutral environment is about 60–70 W/m^2^ according to ISO 8996 [25]. In hot conditions, a maximum increase of 5 to 10 W/m^2^ may be expected due to increased heart rate and sweating [25]. Considering a hot and humid environment in the refuge chamber and the anxious mood of miners while waiting for rescue, a metabolic rate of 80 W/m^2^ was adopted in our heat stress evaluation. A clothing insulation of 0.6 clo was adopted for conservative consideration though miners can strip down to minimal clothing. The air velocity in a refuge chamber is 0.15 m/s. A standard subject with the height of 1.8 m and weight of 75 kg was evaluated. The subject is assumed to be heat unacclimatized but can drink freely.

The mean radiant temperature in a movable refuge chamber is approximately equal to the internal surface temperature of the chamber wall, which can be calculated by Equation (12) under steady state heat transfer conditions:

(12) $$t_{r}=t_{a}+\frac{t_{o}-t_{a}}{h_{i}}K$$

where *t_o_* is the temperature of ambient air outside the refuge chamber, in °C. *K* is the overall heat transfer coefficient of the wall of the refuge chamber, in W/m^2^-K. *h_i_* is the convective heat transfer coefficient at the inner wall surface of the chamber, in W/m^2^-K, which is about 8.7 W/m^2^-K. According to the experimental results of Wang *et al.* [27], the overall heat transfer coefficient of the chamber wall is about 1.5 W/m^2^-K.

## 3. Results and Discussion

**Figure 2:**
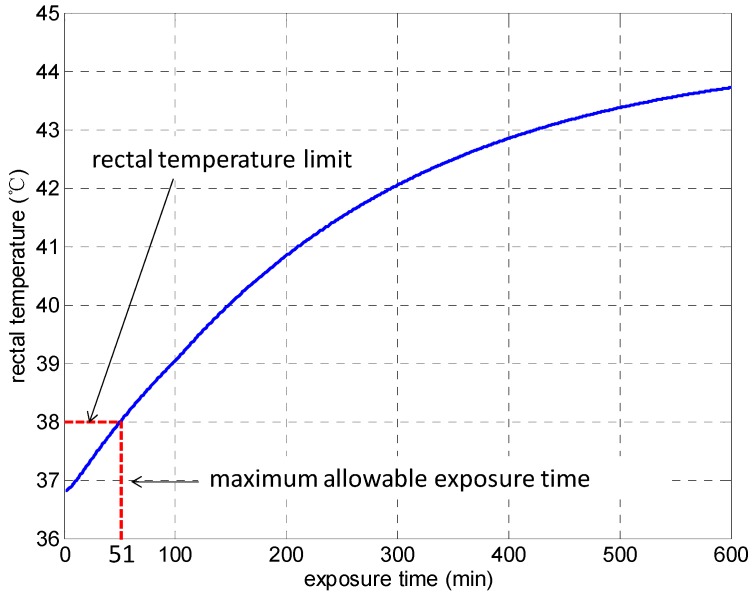
Variation of rectal temperature with exposure time.

**Figure 3:**
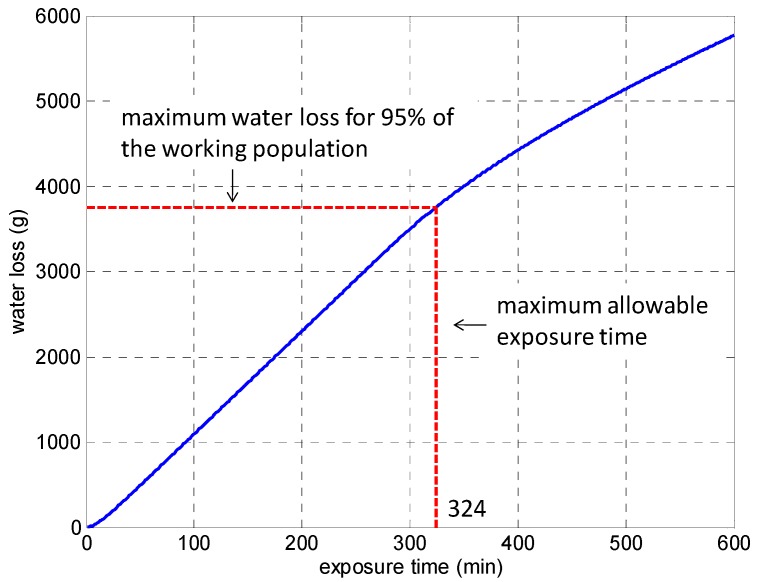
Variation of water loss with exposure time.

The heat stress experienced by trapped miners in a refuge chamber can be analytically evaluated by using the PHS model if the air temperature (*t_a_*) and the relative humidity (*RH*) inside the refuge chamber and the air temperature (*t_o_*) outside the refuge chamber are known. As an example, Figure 2 and Figure 3 respectively present the variations of rectal temperature and water loss with the exposure time of a human body in a refuge chamber for *t_a_* = 35 °C, *RH* = 85%, and *t_o_* = 55 °C, which is required by the early design standard for movable refuge chambers in China.

Figure 2 shows the increases of rectal temperature of trapped miners with the exposure time in a refuge chamber. The rectal temperature may reach 38 °C at the 51st minute and the temperature limit will be exceeded when further exposed in the hot environment, which may result in heat illnesses among trapped miners.

Figure 3 displays the variation of the water loss of the body with exposure time. The water loss increases with exposure time and it reaches the maximum allowable water loss at the 324th minute. The maximum allowable water loss is 3750 g for a subject with a weight of 75 kg, which is 5% of body mass. Further exposure may result in severe dehydration of human body. It can be found that there exists severe heat stress under the given thermal environmental conditions. To simultaneously meet the heat strain criteria for rectal temperature and water loss, the maximum allowable exposure time is 51 min, otherwise it may do harm to the subject.

Table 1 shows the variations of heat stress with the temperature and humidity inside the refuge chamber for *t_o_* = 55 °C. It can be found from Table 1 that both the duration limit of exposure for dehydration (DLE_DH) and the duration limit of exposure for heat storage (DLE_HS) decrease with the increases of air temperature and humidity. However, the DLE_HS is lower than that of DLE_DH, which indicates the heat accumulation is the dominant factor of heat strain under the thermal environmental conditions in a refuge chamber. The lower value of DLE_DH is due to a quick rise in core temperature, which is also illustrated by Figure 2. From Table 1, it can be seen that there exists heat stress, even severe heat stress, in the refuge chamber when the air temperature exceeds 30 °C. The investigation result of Kielblock *et al.* [22] shows that the wet-bulb temperature of the air in a refuge chamber quickly climbed up from 20.9 °C to 35 °C in 90 min after the failure of the compressed air. Thus, refrigerative air conditioning is strongly recommended for refuge chambers to avoid the excessive heat stress and the development of heat illnesses for miners trapped inside [15].

Table 2 and Table 3 display the impacts of air temperature outside the refuge chamber (*t_o_*) and overall heat transfer coefficient of the wall of the refuge chamber on the heat stress inside the refuge chamber, respectively. It can be seen that the duration limit of exposure decreases with the increase of air temperature outside the refuge chamber. Thus, a lower outside air temperature is useful for the survival of workers trapped in the refuge chamber. It is a pity that high temperatures often follow disasters, especially fire, gas and/or dust explosions, which are the most frequently occurring disasters in underground coal mines in China [3]. The air temperature outside the refuge chamber greatly affects the heat stress when the heat stress in the chamber is low. However, the outside air temperature has little impact on the inside heat stress when the heat stress inside chamber is high.

The impact of the overall heat transfer coefficient of the chamber wall on the heat stress inside the chamber is similar to that of the outside air temperature. When the heat transfer coefficient increases, the heat stress in the refuge chamber also increases, however, the maximum allowable exposure time decreases. The impact of the heat transfer coefficient of the chamber wall on the inside heat stress is more obvious under low heat stress condition than under higher one. Therefore, enhancing the heat isolation level of the chamber wall will benefit the indoor thermal environment of the refuge chamber. It can also reduce the rate of heat transferred from outside of the chamber and thus reduce the cooling energy consumption for the inside air conditioning.

The primary purpose of an underground mine refuge chamber is to provide a safe haven for miners working in the immediate area in the event of the atmosphere becoming irrespirable. The heat will build up quickly in the refuge chamber when a number of trapped miners are inside the restricted space if no air-conditioning system is operated. The humidity ratio in the refuge chamber will also increase with the operation time due to the breathing and sweating of occupants. High levels of heat and humidity is harmful or even dangerous, which can cause sweating, paleness, cramps, tiredness, weakness, dizziness, headache, nausea or vomiting, and fainting [28]. Therefore, refrigerative equipment for temperature and humidity control is a fundamental requirement for a refuge chamber [19,29]. Otherwise, excessive heat stress may result in fatalities and injuries, even if the toxic gas concentration in the chamber is at low level.

For temperature and humidity control of a refuge chamber, refrigeration technology and equipment is not a challenge and several options for cooling of the refuge chamber were recommended by Brake *et al.* [30]. Recently, Yang *et al.* [29] and Jia *et al*. [19] already investigated the feasibilities of open-cycle carbon dioxide refrigerators and ice storage capsules for temperature and humidity control of coal mine refuge chambers, respectively. The challenge that the refuge chamber designers face is how to identify the thermal tolerability and the temperature and humidity criteria for maintaining a tolerable thermal environment within a refuge chamber. Of course, if the thermal environment inside the refuge chamber is maintained at a very comfortable level, e.g., dry-bulb temperature of 25 °C and relative humidity of 55%, miner will suffer no heat-related injuries. However, the lower the inside air temperature and humidity level are, the more cooling energy will be consumed, which also means a larger volume and higher initial investment and maintenance costs of the refuge chamber. During an emergency, disconnection from external services is possible for refuge chambers due to damage to electricity, compressed air, and water supply systems caused by the disaster. Under these circumstances, backup power must be available. However, the backup power is limited and air scrubber units, lighting, air conditioning and electronic control systems all require backup power. The duration of independent power sources will be shortened if the air conditioning system consumes too much power. Refuge chambers should be used only under critical circumstances, where the challenge is not the risk of accident, occupational disease, or fitness for work, but far more importantly, saving lives in a catastrophic emergency. Under these circumstances, the priorities are to prolong the duration of energy supplies and shelter operations [7]. Therefore, the temperature and humidity criteria for refuge chamber design should be the upper limit values, under which the trapped miners can safely survive without any injury during the normal protection time.

**Figure 4:**
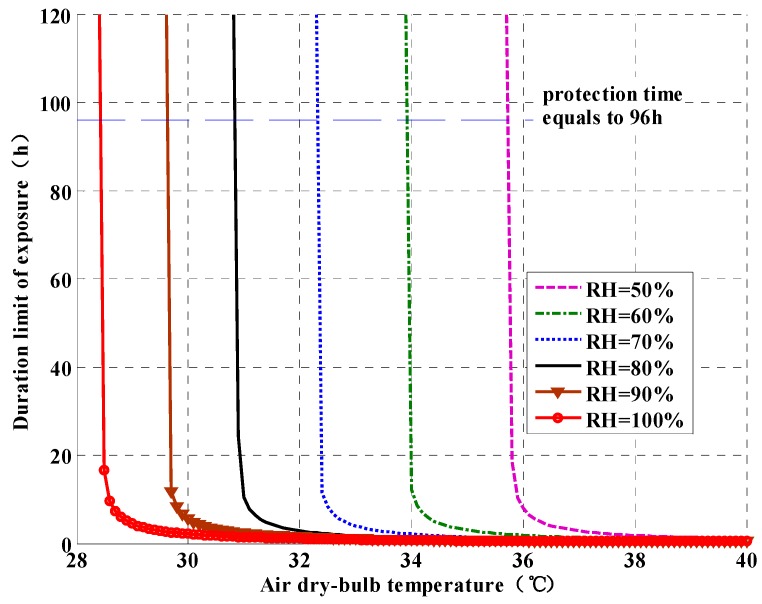
The relationship between the duration limit of exposure and the air temperature and humidity.

To determine the upper limits of temperature and humidity inside a refuge chamber, the PHS model is used to analyze the relationship between the duration limit of exposure(the minimum value of DLE_DH and DLE_HS) and the air temperature and humidity, which is shown in Figure 4 (for *t_o_* = 55 °C and *K* = 1.5 W/m^2^-K). It can be seen that the duration limit of exposure will decrease quickly with the increase of air temperature at a given relative humidity. Similarly, the duration limit of exposure will decrease with the increase of relative humidity under a given dry-bulb temperature. From Figure 4, one can determine the upper limits of internal thermal environment when the required protection time of refuge chamber is given. For example, if the required protection time is 96 h, the internal air temperature should be controlled at 32.3 °C when the relative humidity is controlled at 70%. Otherwise, the required protection time cannot be achieved.

Table 4 presents the upper limits of temperature and humidity for protection time of 96 h. From Table 4, at higher relative humidity the air temperature needs to be lower. The maximum humidity level recommended by Mejías *et al.* [7] was 70.0% inside the confined spaces with air temperature over 30 °C. Therefore, the thermal environment in a refuge chamber is recommended to be controlled at air temperature of 32 °C and relative humidity of 70%.

## 4. Conclusions

The predicted heat strain model was used to quantitatively evaluate the heat stress in a movable refuge chamber. There is severe heat stress if the air temperature in the refuge chamber is higher than 30 °C and appropriate air-conditioning measures should be taken to protect the miners trapped in the refuge chamber from heat injuries. The simulation results show that heat accumulation is the dominant factor that affects the heat strain in a refuge chamber. The impact of air temperature outside the refuge chamber and thermal insulation of the wall of the refuge chamber on heat stress inside the refuge chamber was also investigated. The heat stress in the refuge chamber increases with the increase of the outside air temperature and the decrease of thermal resistance of the refuge chamber wall. Thus, a chamber wall with good thermal insulation is useful to create a good thermal environment in the refuge chamber when the air temperature outside refuge chamber is high. The air temperature and humidity in the refuge chamber are the two dominant factors of the internal heat stress. However, to prolong the duration of energy supplies and shelter operations, the internal thermal environment should be maintained below the upper limit. Therefore, an air temperature of 32 °C and relative humidity of 70% is recommended as the design standard for the internal thermal environment of the movable refuge chamber.

## Figures and Tables

**Table 1:** Duration limit of exposure (min) for various air temperature and humidity inside the refuge chamber.

Relative Humidity (RH)		Air Temperature *t_a_* (°C)					
		30	32	34	36	38	40
**65%**	DLE_DH	WL	WL	WL	363	325	320
	DLE_HS	WL	WL	200	83	53	39
**75%**	DLE_DH	WL	WL	392	329	320	319
	DLE_HS	WL	322	94	56	40	32
**85%**	DLE_DH	WL	462	339	321	319	318
	DLE_HS	WL	119	63	43	33	31
**95%**	DLE_DH	WL	363	322	321	319	318
	DLE_HS	181	75	48	36	33	31

WL, without limit.

**Table 2:** The duration limit of exposure (min) for various air temperatures outside the refuge chamber (*t_o_*) and inside the refuge chamber (*t_a_*).

Air Temperature Inside Refuge Chamber		*t_o_* (°C)				
		20	30	40	50	60
*t_a_* = 35 °C	DLE_DH	337	332	328	325	323
	DLE_HS	63	59	56	53	50
*t_a_* = 33 °C	DLE_DH	WL	446	410	384	366
	DLE_HS	122	108	96	86	78
*t_a_* = 31 °C	DLE_DH	WL	WL	WL	WL	WL
	DLE_HS	WL	WL	405	255	190

**Table 3:** The duration limit of exposure (min) for various overall heat transfer coefficient of the wall of refuge chamber (K) and air temperature inside refuge chamber (*t_a_*).

Air Temperature Inside Refuge Chamber		K (W/m^2^-K)				
		0.5	1	1.5	2	2.5
*t_a_* = 35 °C	DLE_DH	328	326	324	323	322
	DLE_HS	55	53	51	49	48
*t_a_* = 33 °C	DLE_DH	409	389	374	362	353
	DLE_HS	95	88	82	77	72
*t_a_* = 31 °C	DLE_DH	WL	WL	WL	WL	WL
	DLE_HS	432	286	217	177	150

**Table 4:** The upper limits of temperature and humidity for protection time of 96 h.

RH	50%	60%	70%	80%	90%	100%
*t_a_* (°C)	35.7	33.9	32.3	30.8	29.6	28.4
